# Supplementary material for: Characterization and Evaluation of Rapamycin-Loaded Nano-Micelle Ophthalmic Solution
Source: J Funct Biomater. 2023 Jan 16;14(1):49. doi: 10.3390/jfb14010049 (PMC9862165; doi:10.3390/jfb14010049)
Supplement: Supplementary file 1 [file jfb-14-00049-s001.zip › jfb-2059840-supplementary.pdf]

Supplementary Materials

Characterization and Evaluation of Rapamycin-Loaded Nano-micelle Ophthalmic Solution

**Ting Zhang [1,2,3,†], Chao Wei [2,†], Xianggen Wu [4], Sai Zhang [2], Fangnan Duan [2], Xiaolin Qi [1,2,3], Weiyun Shi [1,2,3,*]
and Hua Gao [1,2,3,*]**

1 Eye Hospital of Shandong First Medical University (Shandong Eye Hospital), Eye Institute of Shandong First Medical University, Jinan 250021, China
2 State Key Laboratory Cultivation Base, Shandong Provincial Key Laboratory of Ophthalmology, Eye Institute of Shandong First Medical University, Qingdao 266071, China
3 School of Ophthalmology, Shandong First Medical University, Jinan 250021, China
4 College of Chemical Engineering, Qingdao University of Science and Technology, Qingdao 266101, China
* Correspondence: wyshi@sdfmu.edu.cn (W.S.); hgao@sdfmu.edu.cn (H.G.)
† These authors contributed equally to this work.

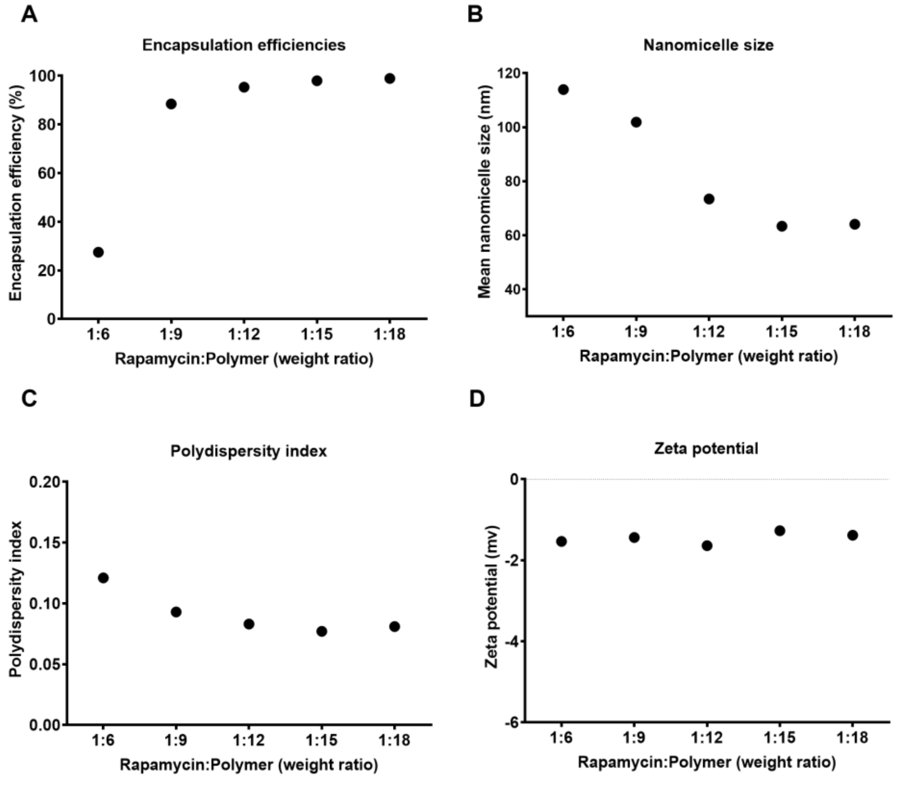

Figure S1. Characteristics of the RAPA-NM consisting of different weight ratios of PVCL-PVA-PEG/RAPA. (A) Encapsulation efficiencies, (B) nano-micelle size, (C) PDI, and (D) zeta potential.

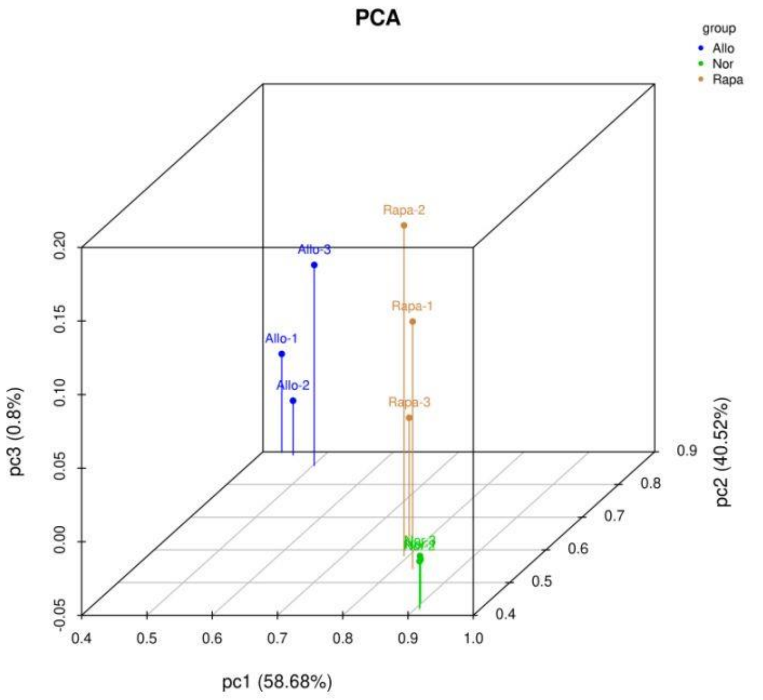

Figure S2. The PCA analysis. The samples obtained from the Nor, Allo and RAPA groups were separated into three clusters in the PCA image.

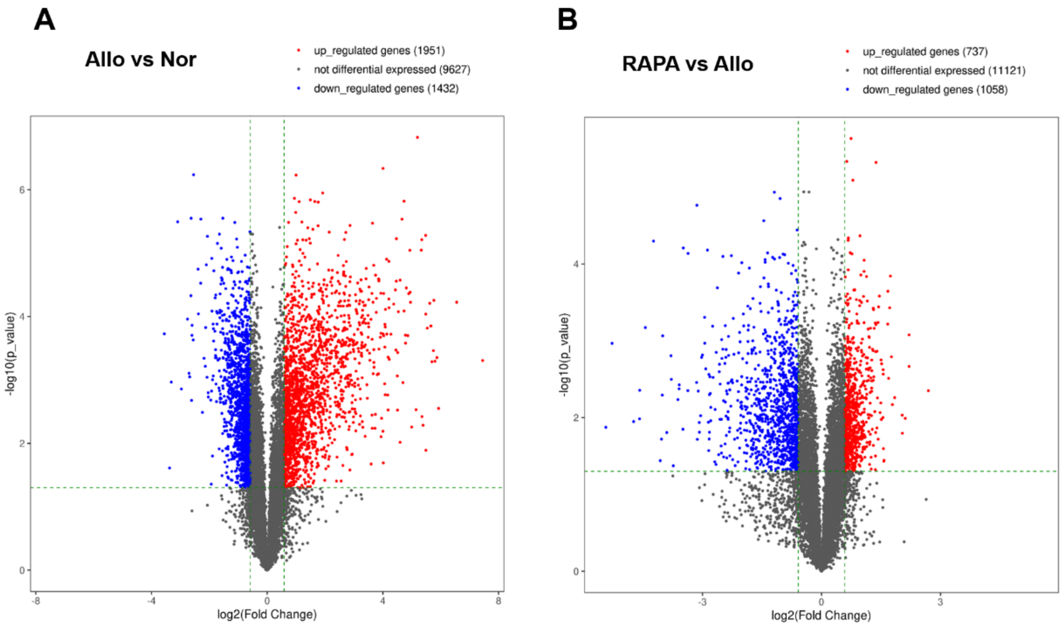

Figure S3. Number of DEGs in the cornea with a value of $p < 0.05$ as shown in a volcano map. (A) Allo vs. Nor group. (B) RAPA vs. Allo group.
